# Präventionsdilemma auf kommunaler Ebene? Einflussfaktoren auf die Teilnahme von Kommunen an Maßnahmen zur Gesundheitsförderung

**DOI:** 10.1007/s11553-022-00964-y

**Published:** 2022-07-05

**Authors:** Annika Herbert-Maul, Karim Abu-Omar, Maike Till, Tobias Fleuren, Andrea R. Wolff, Anne K. Reimers

**Affiliations:** 1 Department für Sportwissenschaft und Sport, Friedrich-Alexander Universität Erlangen-Nürnberg, Gebbertstr. 123b, 91058 Erlangen, Deutschland; 2 Koordinierungsstelle gesundheitliche Chancengleichheit, Landeszentrale für Gesundheit in Bayern, München, Deutschland

**Keywords:** Kommunale Gesundheitsförderung, „Community readiness“, Sozioökonomische Deprivation, Förderprogramme, Partizipation, Community-based health promotion, Community readiness, Socioeconomic deprivation, Funding programs, Participation

## Abstract

**Hintergrund:**

Von Gesundheitsförderung profitieren häufig vorwiegend sozial privilegierte Personen während sozial Benachteiligte seltener erreicht werden. Ob dieses Präventionsdilemma auch auf kommunaler Ebene besteht, wurde bislang kaum erforscht.

**Ziel der Arbeit:**

Die Studie untersucht anhand von zwei bayernweiten Projektausschreibungen zur kommunalen Bewegungsförderung, ob sozioökonomisch deprivierte Kommunen, die geringe Angebotsstrukturen der Gesundheitsförderung aufweisen, durch solche Ausschreibungen erreicht werden und welche Faktoren die Bereitschaft zur Beteiligung beeinflussen.

**Methoden:**

Es werden Bevölkerungsdaten und sozioökonomische Deprivationsdaten von (*n* = 171) Kommunen aus drei Gruppen (teilnehmende/interessierte Kommunen und deprivierte Vergleichskommunen) verglichen. Durch eine systematische Internetrecherche werden die Gesundheitsförderungsaktivitäten ausgewählter Kommunen erhoben. Barrieren und Förderfaktoren für die Umsetzung der Projekte werden mittels einer Dokumentenanalyse erfasst.

**Ergebnisse:**

Die teilnehmenden und interessierten Kommunen weisen höhere Einwohnerzahlen und geringere Deprivationswerte als der Durchschnitt der Kommunen in Bayern auf und stellen mehr Angebote der Gesundheitsförderung bereit als deprivierte Vergleichskommunen. Großen Einfluss auf die Teilnahme an den Projekten haben finanzielle Faktoren, politische Unterstützung und engagierte Personen.

**Diskussion:**

Die Ergebnisse dieser Studie deuten auf ein Präventionsdilemma auf kommunaler Ebene hin. Um gesundheitlichen Ungleichheiten zwischen Kommunen vorzubeugen, sollten benachteiligte Kommunen in die Gestaltung von Förderprogrammen einbezogen werden, um diese Programme an deren Bedürfnisse anzupassen.

## Abstract (en)

**Background:**

Health promotion often benefits mainly socially privileged persons, while disadvantaged people are reached less often. It remains an important question whether the same holds true for the recruitment of communities to take part in health promotion projects.

**Objectives:**

Based on two Bavarian projects for community-based physical activity promotion, this study examines whether socioeconomically disadvantaged communities with low health promotion structures are being reached to apply for project funding and which factors influence their readiness to participate.

**Methods:**

Data on population level indicators and socioeconomic disadvantages of 171 communities are compared for participating/interested communities and disadvantaged communities. By means of a systematic internet search, health promotion activities of selected communities are analyzed. Barriers and facilitating factors for the implementation of the two projects are recorded through a document analysis.

**Results:**

The participating and interested communities have larger populations, are less disadvantaged, and provide more health promotion offers than disadvantaged communities. Financial factors, political support, and committed leadership have a large influence on participation.

**Discussion:**

The results of this study point to an inequality paradox for preventive actions on the community level. In order to prevent health inequalities between communities, disadvantaged communities might need to be involved in the design of funding programs in order to tailor these programs to their needs and thus make it easier for them to acquire project funding.

## Einleitung

Kommunen (d. h. Landkreise, Städte, Gemeinden etc.) sind als Setting für die Gesundheitsförderung von sozial benachteiligten Personen von großer Bedeutung [15]. In Kommunen können Angebote zur Gesundheitsförderung in den Lebenswelten der adressierten Personen umgesetzt und Wohn- bzw. Lebensbedingungen, insbesondere in benachteiligten Stadtgebieten, gesundheitsförderlich gestaltet werden. Durch das Gesetz zur Stärkung der Gesundheitsförderung und Prävention wurde die Bedeutung von lebensweltbezogenen Maßnahmen betont. Die Umsetzung entsprechender Maßnahmen wurde durch die Verpflichtung der gesetzlichen Krankenversicherungen (GKV), einen höheren Anteil der Einnahmen aus den Versicherungsbeiträgen für lebensweltbezogene Maßnahmen der Gesundheitsförderung und Prävention auszugeben, gestärkt [4]. Dementsprechend entwickelten das GKV-Bündnis für Gesundheit und Krankenkassen Förderprogramme zur Finanzierung kommunaler Gesundheitsförderungsmaßnahmen.

Auf Ebene der Individuen zeigt sich, dass durch gesundheitsfördernde Maßnahmen vorwiegend Personen erreicht werden, die aufgrund ihres gesunden Lebensstils und ihrer Lebenslage tendenziell geringere Risikofaktoren für Erkrankungen aufweisen [10, 13]. Dieses Phänomen, oft als „Präventionsdilemma“ bezeichnet, konsolidiert gesundheitliche Chancenungleichheiten und stellt eine große Herausforderung der Gesundheitsförderung dar [1, 10, 13]. Es wird angenommen, dass eine ähnliche Problematik auch auf kommunaler Ebene vorliegt [16], doch dies wurde bislang nicht empirisch untersucht. Folglich ist nicht bekannt, ob sozioökonomisch bessergestellte Kommunen, die ohnehin vielfältige Maßnahmen der Gesundheitsförderung umsetzen, häufiger öffentliche Projektmittel für Gesundheitsförderung beantragen als sozioökonomisch deprivierte Kommunen.

In dieser Fallstudie wird untersucht, inwiefern Kommunen, die zwei ausgewählte Projekte der Gesundheitsförderung (BIG(Bewegung als Investition in Gesundheit)-Projekt und GESTALT[GEhen, Spielen, Tanzen Als Lebenslage Tätigkeit]-Projekt) umsetzen, sich von anderen Kommunen unterscheiden. Der Vergleich dieser Kommunen erfolgt anhand von Untersuchungsparametern, die aus dem Modell zur „community readiness“ (CR) nach Edwards et al. [5] abgeleitet wurden. Das CR-Modell beschreibt, dass die (Handlungs‑)Bereitschaft einer Kommune zur Ergreifung von gesundheitsförderlichen Maßnahmen („community readiness“) u. a. von lokalem Wissen über bzw. bestehenden Präventionsmaßnahmen, verfügbaren Ressourcen und einer Führung („leadership“) abhängig ist [5, 7].

Die Analyse soll die Frage beantworten, ob durch niederschwellige Projektausschreibungen jene Kommunen erreicht werden, die eine hohe sozioökonomische Deprivation aufweisen. Außerdem wird erforscht, welche Faktoren die Teilnahme an Maßnahmen der Gesundheitsförderung beeinflussen.

## Methode

### BIG und GESTALT als Fallstudien

Die Projekte dieser Fallstudie (BIG & GESTALT) wurden an der Friedrich-Alexander Universität Erlangen-Nürnberg (FAU) entwickelt und verfolgen das Ziel, mittels eines partizipativen Ansatzes kommunale Strukturen der Bewegungsförderung aufzubauen, um das Bewegungsverhalten von Frauen in schwierigen Lebenslagen (BIG-Projekt) bzw. Personen ab 60 Jahren mit einer erhöhten Prädisposition für demenzielle Erkrankungen (GESTALT-Projekt) zu fördern. BIG wurde 2005 entwickelt und seitdem auf 16 deutsche Standorte übertragen, GESTALT startete 2012 und seitdem an fünf Standorten umgesetzt. Eine ausführliche Beschreibung der Projekte findet sich hier [12, 17].

### BIG-5 und GESTALT – Get 10-Projektausschreibung

Um die Verbreitung von BIG und GESTALT in Bayern voranzutreiben, sollen die Projekte auf 15 neue Kommune übertragen werden (BIG: *n* = 5/GESTALT: *n* = 10). Diese Kommunen erhalten die Möglichkeit, eines der beiden evidenzbasierten, evaluierten und praxiserprobten Projekte mit Hilfe einer umfangreichen Förderung umzusetzen. Die Umsetzung wird für 3,5 Jahren von Wissenschaftler*innen der FAU unterstützt und wissenschaftlich begleitet. Die Förderung erfolgt durch die BZgA mit Mitteln der gesetzlichen Krankenkassen im Rahmen des GKV-Bündnisses für Gesundheit (www.gkv-buendnis.de). Zur Teilnahme müssen die Kommunen eine Interessensbekundung unterzeichnen und einen geringen finanziellen Eigenanteil leisten. Durch diese Vorgehensweise wird eine niederschwellige Projektbeteiligung für die Kommunen gewährleistet. Die Projektkonzeption entfällt für die Kommunen.

Zur Akquise der 15 neuen Kommunen wurden die Projekte über Newsletter sowie über die Webseiten der Projekte (www.big-projekt.de; www.gestalt-kompetenzzentrum.de) und der Landeszentrale für Gesundheit in Bayern (www.lzg-bayern.de) beworben. Zudem wurden die Förderprogramme in sozialen Medien veröffentlicht und einzelne Kommunen gezielt über die Förderprogramme informiert.

### Stichprobe

Es werden drei Untersuchungsgruppen miteinander verglichen:

#### „Teilnehmende Kommunen“:

*N* = 27 bayerische Kommunen; darunter 15 Kommunen, in denen eines der Projekte bereits vor der neuen Ausschreibung umgesetzt wurde und 14 „neue“ Kommunen, die eines der Projekte im Rahmen der neuen Förderung umsetzen (2 Städte zählen zu den neuen und zu den langjährigen Projektstandorten).

#### „Interessierte Kommunen, die nicht teilnehmen“:

*N* = 23 Kommunen, die Interesse an dem Förderprogramm zeigten, die Projekte jedoch nicht umsetzen.

#### „Deprivierte Vergleichskommunen“:

*N* = 121 Kommunen, die aufgrund ihrer sozioökonomischen Deprivation zum untersten Quintil der Kommunen in Bayern zählen (= GISD-Dezentil > 8).

Die dritte Gruppe dient als Vergleich, um zu erforschen, inwiefern sich deprivierte Kommunen von den teilnehmenden bzw. interessierten Kommunen unterscheiden. Es werden nur Städte und Landkreise mit mehr als 5000 Einwohner*innen einbezogen, da die Ausschreibung primär an entsprechende Kommunen gerichtet war.

### Datensammlung und -auswertung

Die Studie basiert auf einen Mixed-method-Ansatz (tabellarische Zusammenfassung der angewendeten Methoden im Anhang):

1. Quantitative Dokumentenanalyse

Anhand von Berichten des bayerischen Landesamts für Statistik sowie Ergebnissen des Zensus 2011 werden quantitative Daten zu folgenden Merkmalen der Kommunen (*n* = 171) gesammelt: a) Größe der Kommune, b) Anteil an Personen mit Migrationshintergrund, c) Altersdurchschnitt und d) Stadt oder Landkreis. Zudem werden Daten zum Deprivationsgrad der Kommunen mittels des German Index of Socioeconomic Deprivation (GISD) erhoben. Der GISD bildet auf einer Skala von 0–1 den Grad der sozioökonomischen Deprivation für Gemeinden, Kreise und Regierungsbezirke ab (0 beziffert den niedrigsten und 1 den höchsten Deprivationsgrad). Mithilfe des GISD-Scores werden die Dezentile (GISD-10) errechnet (1 = niedrigste Deprivation; 10 = höchste Deprivation; [9]). Für die vorliegenden Analysen werden die Daten auf Gemeindeebene herangezogen.

2. Webbasierte Inhaltsanalyse

Die Aktivität der Kommunen im Bereich der Gesundheitsförderung wird anhand einer systematischen Internetrecherche (in Anlehnung an McMillan [11]) erfasst. Zunächst wird analysiert, ob die Kommunen durch das Konzept „Gesundheitsregionen^plus^“ des Freistaats Bayern gefördert werden (Fördermittel u. a. für den Aufbau von Strukturen der Gesundheitsförderung und Prävention) [8]. Zusätzlich wird in vier Praxisdatenbanken (Praxisdatenbank des Kooperationsverbundes gesundheitliche Chancengleichheit, ZPG-Bayern, bayerischen Landesamtes für Gesundheit und Lebensmittelsicherheit und von IN-FORM) sowie auf den Internetseiten der Kommunen und anhand einer freien Internetrecherche mit der Suchmaschine Google nach Gesundheitsförderungsaktivitäten an den Standorten recherchiert (Suchbegriffe „Gesundheit“, „Gesundheitsförderung“, „Prävention“ + jeweils *„Name der Kommune“*). Die Gesamtheit aller ermittelten Projekte, Veranstaltungen, Angebote, Netzwerke, Konzepte und Einrichtungen ergeben die Anzahl der gesundheitsförderlichen Aktivitäten je Standort.

Die Stichprobe der Kommunen für die Webanalyse umfasst *n* = 22 Kommunen (es liegen zwei Überschneidungen vor, da je ein Landkreis und eine Stadt in zwei Untersuchungsgruppen vertreten sind).

3. Qualitative Dokumentenanalyse

Während der Projektausschreibung wurde anhand von Gesprächsnotizen und der Analyse des Schriftverkehrs dokumentiert, welche Faktoren die Antragstellung erleichterten bzw. erschwerten und welche Gründe dazu geführt haben, dass Kommunen trotz anfänglichem Interesse letztlich nicht an der Ausschreibung teilnahmen. Diese Dokumente werden qualitativ ausgewertet und eine Liste der genannten Barrieren und Förderfaktoren erstellt.

## Ergebnisse

1. Analyse der Bevölkerungs- und Sozialdaten

Die Tab. 1 fasst die Bevölkerungs- und Sozialdaten aller untersuchten Kommunen zusammen.

**Tab. 1:** Vergleich von ausgewählten Bevölkerungs- und Sozialdaten der verschiedenen Untersuchungsgruppen

		Kommunen in Bayern (> 5000 Perso﻿n﻿e﻿n)	Teilnehmende Kommunen (neue und langjährige)	Interessierte Kommunen (nehmen nicht teil)	Deprivierte Vergleichskommunen (GISD-Dezentil > 8)
*N*		642	27	23	121
Art der Kommune	Großstädte (> 100.000 Einwohner*innen)	8	6	2	0
	Landkreise	71	7	7	13
	Kleinere Städte & Gemeinden (< 100.000 Einwohner*innen)	563	14	14	108
Bevölkerungsdaten (Median)	Zahl der Einwohner*innen [2]	9313	71.566	42.743	8289
	Anzahl an Einwohner*innen mit Migrationshintergrund^a^ [14]	–^b^	20,9 %	17,9 %	–^b^
	Durchschnittsalter der Einwohner*innen [2]	44,5	44,5	45,3	45,3
Deprivationsgrad	GISD-Score (Median)	0,4598	0,4593	0,4774	0,5216
	GISD-Dezentil (Median)	6	6	7	10
	Niedrigster GISD-Score	0,1476	0,2421	0,2560	0,5022
	höchster GISD-Score	0,6288	0,5346	0,5600	0,6288

*GISD* German Index of Socioeconomic Deprivation

^a^Aktuellste Daten von 2011, Werte könnten inzwischen gestiegen sein

^b^Zu viele fehlende Daten, da Daten erst für Kommunen ab einer Größe von 10.000 Einwohnern vorliegen. Der Anteil der Menschen mit Migrationshintergrund in ganz Bayern liegt bei 19,1 %

Der Großteil der teilnehmenden Kommunen sind Städte und Gemeinden mit bis zu 100.000 Einwohner*innen. Sechs der acht bayerischen Großstädte (> 100.000 Einwohner*innen) nehmen an mindestens einem der beiden Projekte teil. Kleinere Städte, Gemeinden und Landkreise sind unter den interessierten Kommunen etwas und unter den deprivierten Vergleichskommunen deutlich stärker vertreten als unter den teilnehmenden Kommunen. Die mittlere Einwohner*innenzahl der teilnehmenden Kommunen ist höher als die der interessierten Kommunen und mehr als 8‑mal so hoch wie die der deprivierten Vergleichskommunen und aller bayerischen Kommunen. Diese Zahlen zeigen, dass kleinere Gemeinden und Landkreise zwar erreicht werden, jedoch unterrepräsentiert sind, da unter den teilnehmenden Kommunen vorwiegend bevölkerungsstarke Standorte vertreten sind.

Die Mediane der Anzahl an Personen mit Migrationshintergrund sowie des Durchschnittsalters zeigen keine auffallenden Unterschiede zwischen den Untersuchungsgruppen.

Die Mediane des GISD-Scores der teilnehmenden Kommunen und der Gesamtheit aller Kommunen in Bayern unterscheiden sich kaum. Die GISD-Dezentile zeigen, dass die teilnehmenden Kommunen heterogen hinsichtlich ihres Deprivationsgrades sind: die Hälfte der Kommunen (*n* = 14) weisen ein GISD-Dezentil von sechs und höher auf, gelten also als sozial deprivierter als 50 % der Kommunen in Bayern. Besonders durch die neue Projektausschreibung wurden sozial schlechter gestellte Kommunen erreicht. Dies lässt sich am Vergleich der GISD-Dezentile der langjährig teilnehmenden Kommunen (GISD-Dezentil: 5), der neu teilnehmenden Kommunen (GISD-Dezentil: 6) und der interessierten Kommunen (GISD-Dezentil: 7) ablesen.

2. Analyse der Aktivität im Bereich der Gesundheitsförderung

19 der analysierten Kommunen werden als Gesundheitsregion^plus^ gefördert (jeweils eine interessierte und eine teilnehmende Kommune sind nicht in diesem Förderprogramm vertreten; [8]).

Um die Anzahl der gesundheitsförderlichen Aktivitäten (= Angebote, Projekte, Initiativen, Konzepte etc.) der verschiedenen Kommunen zu vergleichen, wurden jeweils die Aktivitäten je 100.000 Einwohner*innen berechnet (Tab. 2). Zwischen Landkreise und Städte/Gemeinden zeigen sich deutliche Unterschiede. So liegt der Median der Anzahl der gesundheitsförderlichen Aktivitäten bei den teilnehmenden und interessierten Landkreisen deutlich unter dem Median der analysierten Städte und Gemeinden. Bei den deprivierten Vergleichskommunen zeigt sich diese Unterscheidung nur in geringem Maße. Bei den Städten und Gemeinden ist der Median der gesundheitsförderlichen Aktivitäten der teilnehmenden und interessierten Kommunen gleich und doppelt so hoch wie bei den deprivierten Vergleichskommunen.

3. Barrieren und Förderfaktoren für die Teilnahme an Gesundheitsförderungsprojekten

**Tab. 2:** Anzahl der im Internet aufgeführten gesundheitsförderlichen Aktivitäten ausgewählter Kommunen

	Art der Kommune	Kommune	GISD-Dezentil	Einwohner*innen		Gesundheitsförderliche Aktivitäten	
				Anzahl (2019)	Median	Je 100.000 Einwohner	Median
Teilnehmende Kommunen	Landkreise	Kulmbach	10	71.566	117.853	27,95	7,64
		Fürth	6	117.853		7,64	
		Ostallgäu	5	141.182		4,25	
	Städte/Gemeinden	Selb	9	14.895	127.934	13,43	41,43
		Weiherhammer	10	3831		78,31	
		Würzburg	4	127.934		41,43	
		Regensburg	1	153.094		61,40	
		Fürth	7	128.497		17,12	
Interessenten	Landkreise	Freising	1	180.007	134.573	10,56	4,46
		Neumarkt	3	134.573		4,46	
		Roth	9	126.749		3,94	
	Städte/Gemeinden	Bamberg	5	77.373	14.515	50,41	41,43
		Würzburg	4	127.934		41,43	
		Pressath	9	4271		46,83	
		Eckental	1	14.515		34,45	
		Furth im Wald	10	9084		11,01	
Deprivierte Vergleichskommunen	Landkreise	Schweinfurt	10	115.445	77.410	9,53	16,79
		Kulmbach	10	71.566		27,95	
		Regen	10	77.410		16,79	
	Städte/Gemeinden	Hof	10	45.825	5685	39,28	19,70
		Neustadt a.d. Waldnaab	10	5685		87,95	
		Friedberg	10	29.979		13,34	
		Leutershausen	10	5633		17,75	
		Weitramsdorf	10	5076		19,70	

Die Ergebnisse der Analyse der Barrieren und Förderfaktoren für die Teilnahme an den Projekten sind in Tab. 3 und 4 zusammengefasst.

**Tab. 3:** Barrieren bei der Beantragung und Umsetzung von Projekten

Barriere	Erläuterung
Finanzielle Ressourcen	Die Erbringung eines Eigenanteils stellte für einige Kommunen eine Hürde dar. Teilweise war die Planung des Haushaltsjahres bereits abgeschlossen und somit keine finanziellen Mittel verfügbar
(Entscheidungs‑)Strukturen und Zeit	Die Bewilligung eines Projekts durch kommunale Entscheidungsgremien sowie die administrative Vorbereitung der Projekte (z. B. Einrichtung einer Koordinationsstelle) benötigen häufig viel Zeit (bis zu einem Jahr). Aufgrund der begrenzten Förderdauer musste die Entscheidung für die Umsetzung des Projekts innerhalb weniger Monate getroffen werden
COVID-19-Pandemie	Die Projektausschreibung erfolgte vom Winter 2020 bis Frühjahr 2021. In dieser Zeit waren viele kommunale Verwaltungen vorwiegend mit der Bekämpfung der COVID-19-Pandemie beschäftigt, sodass keine finanziellen, zeitlichen und personellen Ressourcen für neue Projekte vorhanden waren
Fehlende Projektkoordination	An einzelnen Standorten konnte keine Person gefunden werden, die die Koordination des Projekts übernommen hätte
Fehlende politische Unterstützung	In mehreren Kommunen konnte keine politische Unterstützung gewonnen werden, da andere Themen bzw. Adressatengruppen größere Prioritäten hatten
Verankerung des Projekts in Landkreisen	In einigen Landkreisen stellte es eine Hürde dar, dass sich keine Kommune bzw. Behörde im Landkreis fand, bei der das Projekt angesiedelt werden konnte
Förderstruktur	Als Antragstellende konnten nur Kommunen auftreten. Die Antragstellung durch nicht städtische Einrichtungen, z. B. Wohlfahrtsverbände, war nicht vorgesehen. Meldeten sich Interessenten von solchen Einrichtungen, so musste zunächst eine kommunale Einrichtung gefunden werden, bei der das Projekt formal angesiedelt werden konnte^a^

*COVID-19* „coronavirus disease 2019“

^a^Da dies eine große Barriere darstellte, wurde eine Ausnahmeregelung vereinbart, nach der auch nicht-städtische Einrichtungen Antragstellende sein können, solange die Kommune das Projekt ebenfalls unterstützt

**Tab. 4:** Förderfaktoren bei der Beantragung und Umsetzung von Projekten

Förderfaktor	Erläuterung
Unterstützung durch politische Entscheidungstragenden	Die Unterstützung von politischen Entscheidungstragenden und Netzwerkpartner*innen ist insbesondere förderlich, um die Projekte in kommunalen Strukturen zu verankern und um finanzielle Mittel für deren Umsetzung zu gewinnen
Vorhandene Strukturen	Als förderlich haben sich Strukturen, wie Netzwerke, vorhandene Leitlinien (z. B. Integrationskonzept oder seniorenpolitisches Gesamtkonzept), Vereine und ein*e Beauftragte*r für Gesundheit erwiesen. Diese Strukturen können Prozesse (z. B. die Suche nach Kooperationspartner*innen) erleichtern und beschleunigen
Finanzielle Mittel	Die Möglichkeit zur kurzfristigen Bereitstellung kommunaler Gelder zur Leistung des finanziellen Eigenanteils erwies sich als förderlich
Niederschwellige Antragstellung	Dass im Rahmen dieser Projektausschreibung nur eine Interessensbekundung notwendig war, erwies sich als hilfreich. Dadurch wurde die ressourcenintensive Antragstellungen umgangen

## Diskussion

Ein wesentliches Ergebnis dieser Studie ist, dass vorwiegend größere Städte an BIG und GESTALT teilnehmen bzw. interessiert sind, während Kommunen mit niedrigeren Einwohner*innenzahlen (ca. < 40.000 Einwohner*innen) seltener Interesse zeigen. Der Deprivationsgrad der erreichten Kommunen ist heterogen. Es werden sowohl sozioökonomisch bessergestellte als auch schlechter gestellte Kommunen erreicht. Dennoch liegt der Median des Deprivationsgrades der teilnehmenden und interessierten Kommunen über dem bayerischen Durchschnitt. Ein Grund dafür könnte in der Größe der erreichten Kommunen liegen, da insbesondere kleinere Städte häufig sozioökonomisch depriviert sind [9] und diese bislang selten erreicht werden. Die Analyse der gesundheitsförderlichen Aktivitäten zeigt, dass teilnehmende und interessierte Städte deutlich mehr Maßnahmen zur Förderung der Gesundheit ihrer Einwohner*innen bieten (absolut und relativ) als Kommunen der Vergleichsgruppe bzw. die analysierten Landkreise.

Als wesentliche Faktoren für die Beantragung und Umsetzung der Projekte haben sich politische Unterstützung und das Engagement einzelner Personen herausgestellt. Einen großen Einfluss hat zudem die Verfügbarkeit von finanziellen Ressourcen. Dies beinhaltet sowohl die kurzfristige Bereitstellung von kommunalen Geldern als auch die niederschwellige Beantragung von Projektfördermitteln.

### CR als Einflussfaktor auf die Projektteilnahme

Mehrere Faktoren, die in der Literatur als ausschlaggebend für die Bereitschaft einer Organisation für Veränderung beschrieben werden [5, 7], stellen sich auch in dieser Studie als relevant für die Handlungsbereitschaft der Kommune heraus (Handlungsbereitschaft wird in dieser Untersuchung angenommen, wenn eine Interessensbekundung oder Teilnahme an den Projekten vorliegt). So ist anzunehmen, dass die teilnehmenden und interessierten Kommunen über mehr Kenntnisse zur Umsetzung gesundheitsfördernder Maßnahmen verfügen, da diese eine größere Anzahl an gesundheitsförderlichen Aktivitäten anbieten. Auch zeigt sich ein leichter Zusammenhang zwischen den finanziellen Ressourcen der Kommunen (gemessen am GISD-Wert) und deren Handlungsbereitschaft. Zudem erweist sich eine engagierte Person und deren Fähigkeit zur Gewinnung politischer Unterstützung als Förderfaktor, bzw. das Fehlen einer solchen Person als Barriere. Darüber hinaus stellen sich situative Faktoren (z. B. COVID-19-Pandemie [„coronavirus disease 2019“], abgeschlossene Planung des Haushaltsjahres) als Barrieren für die Projektumsetzung dar.

In künftigen Studien müssten auch weitere Untersuchungsparameter herangezogen werden, um die Handlungsbereitschaft von Kommunen besser einschätzen zu können. Zu diesen Parametern zählen u. a. die Vernetzungsstrukturen vor Ort, die vorhandenen fachlichen Kompetenzen im Bereich der Gesundheitsförderung, Strategien zur Einwerbung von Fördermitteln, Veränderungsbereitschaft der Bewohner*innen, Bedarfs- und Problembewusstsein der Verantwortlichen, Grad der aktiven Beteiligung der Mitglieder und der Bevölkerungsanteil an Menschen in schwierigen Lebenslagen [5, 7, 16]. Bei der Untersuchung dieser Parameter sollten sowohl quantitative Methoden als auch qualitative Methoden (z. B. Interviews mit kommunalen Führungskräften) zum Einsatz kommen.

### Partizipation und Empowerment von Kommunen zur Prävention von gesundheitlicher Ungleichheit

Von Frohlich und Potvin (2008) wurde festgestellt, dass gesundheitsfördernde Maßnahmen individuelle gesundheitliche Ungleichheiten zwischen sozialen Gruppen verstärken, wenn von diesen vorwiegend Personen profitieren, die ohnehin geringe gesundheitliche Risikofaktoren aufweisen [6]. Die Ergebnisse unserer Untersuchung weisen darauf hin, dass auch auf kommunaler Ebene ein ähnliches Präventionsdilemma vorliegen kann. Wenn im Rahmen von öffentlichen Förderausschreibungen vorwiegend Kommunen finanzielle Mittel beantragen und erhalten, die aufgrund bestehender gesundheitsförderlicher Aktivitäten und vorliegender finanzieller Ressourcen ohnehin einen geringeren Bedarf haben, kann dies dazu führen, dass sich gesundheitliche Ungleichheiten zwischen Kommunen verstärken.

Folglich ist von besonderem Interesse, wie die Handlungsbereitschaft deprivierter Kommunen erhöht und Förderprogramme künftig ausgerichtet und beworben werden müssten, um insbesondere Kommunen zu erreichen, die am meisten davon profitieren. Auf individueller Ebene werden Partizipation und Empowerment von sozial Benachteiligten als wirkungsvolle Ansätze zur Reduktion des Präventionsdilemmas erachtet [1, 10]. Ebenso könnte es auf kommunaler Ebene gelingen, Städte und Gemeinden zur Beantragung von Fördergeldern und Umsetzung von Maßnahmen zu motivieren, wenn sie an der Gestaltung von Fördermaßnahmen und Ausschreibungsregularien beteiligt würden. Denkbar wären Fokusgruppen mit Vertretenden von sozial benachteiligten Kommunen, um zu erheben, welche Barrieren ihnen die Fördermittelakquise und Maßnahmenumsetzung erschweren, welche Gesundheitsförderungsthemen durch die jeweiligen Förderprogramme behandelt werden sollten, auf welchen Informationswegen Förderprogramme beworben werden sollten und welche Kapazitäten (finanziell, personell und zeitlich) notwendig wären, um sie zur Beantragung von Fördermitteln und Umsetzung von Maßnahmen zu befähigen. In diese Erhebung sollte zudem die Sichtweise von kleinen Kommunen und Landkreisen einbezogen werden, da diese bei der Umsetzung von Maßnahmen möglicherweise vor spezifischen Herausforderungen stehen.

### Institutionelle Rahmenbedingungen für die Projektteilnahme

Neben der Gestaltung von Förderanträgen, kann davon ausgegangen werden, dass auch institutionelle Rahmenbedingungen die Handlungsbereitschaft von Kommunen beeinflussen. Ein wesentlicher Faktor dürfte hierbei z. B. die Personalkapazitäten der öffentlichen Gesundheitsdienste (ÖGD) sein. Da der ÖGD mit vielfältigen Aufgaben betraut ist (z. B. Gesundheitsberichterstattung, Schuleingangsuntersuchung etc.), sind die zeitlichen Ressourcen der Mitarbeitenden für die Akquise von Projektmitteln und Umsetzung von Gesundheitsförderungsmaßnahmen u. U. begrenzt. Insbesondere in deprivierten Kommunen bleiben so kaum finanzielle Ressourcen für die Umsetzung von Gesundheitsförderung bzw. für den Eigenanteil, der bei Projektförderungen durch Krankenkassen laut SGB V zu leisten ist [3]. Diese Problematik könnte adressiert werden, wenn es gelingt, auch finanziell schlechter gestellte Kommunen darin zu unterstützen, Personalstellen zu schaffen, die es ihnen erlauben, mehr Aufgaben im Bereich der Gesundheitsförderung wahrzunehmen.

### Limitationen und Stärken

Die Projektausschreibung fand zur Zeit des zweiten Lockdowns während der COVID-19-Pandemie statt. Dieser Ausnahmezustand und die daraus resultierenden geringen Personalkapazitäten könnten kommunale Verwaltungen davon abgehalten haben, neue Projekte zu initiieren.

Zudem weist die Web-Analyse der gesundheitsförderlichen Aktivitäten der Kommunen Limitationen auf. Die Qualität der erhobenen Daten ist stark von der Internetpräsenz der Kommunen abhängig. Auch wurde bei der Analyse ausschließlich die Quantität, nicht aber die Qualität der Aktivitäten bewertet (z. B. wurden langfristige Projekte, die viele Personen erreichten, gleichgewichtet wie einmalige Veranstaltungen). Die Validität dieser Analysen ist somit eingeschränkt. Dennoch kann davon ausgegangen werden, dass diese Quantifizierung der gesundheitsförderlichen Aktivitäten eine hinreichende Differenzierung der Kommunen hinsichtlich ihrer Angebote der Gesundheitsförderung erlaubt.

Des Weiteren hätten durch qualitative Interviews mit den Verantwortlichen der teilnehmenden und interessierten Kommunen umfangreichere Ergebnisse bezüglich der Barrieren und Förderfaktoren der Projektteilnahme erzielt werden können. Diese hätte die durch die Dokumentenanalyse gewonnen Daten ergänzen können.

Darüber hinaus wurde in dieser Studie das Vorliegen eines kommunalen Präventionsdilemmas anhand von zwei Projekten und einer begrenzten Anzahl von Kommunen untersucht. Weitere Untersuchungen mit ähnlichen Forschungsfragen, könnten eine höhere Datendichte erzeugen und somit zusätzliche Ergebnisse generieren.

Bislang gibt es kaum Studien, in denen das Konzept des Präventionsdilemmas auf die kommunale Ebene übertragen wurde. Die Ergebnisse dieser Untersuchung leisten damit einen wichtigen Beitrag zum Forschungsstand und können von großer Relevanz sein, um Ungleichheiten zwischen Kommunen vorzubeugen, bzw. diese zu reduzieren.

## Fazit für die Praxis

- Um die Handlungsbereitschaft zur Teilnahme an Gesundheitsförderungsmaßnahmen von deprivierten Kommunen zu erhöhen, könnte deren Partizipation bei der Gestaltung von Förderprogrammen erfolgsversprechend sein. Auf diesem Wege könnten Fördervoraussetzungen bedarfsgerecht gestaltet und dadurch einer gesundheitlichen Benachteiligung der Bevölkerung deprivierter sowie kleinerer und ländlicher Kommunen vorgebeugt werden.
- Darüber hinaus könnten institutionelle Veränderungen wie die Erhöhung von Personalkapazitäten im ÖGD (öffentlichen Gesundheitsdienst) auch depriviertere Kommunen dazu befähigen, mehr Gesundheitsförderung umzusetzen.

## Anhang

**Tab. 5:** Zusammenfassung der Erhebungsmethoden

	Methoden	Untersuchte Dokumente	Zweck der Datenerhebung	Einbezogene Stichprobe
1	Quantitative Dokumentenanalyse	Berichte des bayerischen Landesamts für Statistik, Ergebnisse des Zensus 2011, German Index of Socioeconomic Deprivation (GISD)	Erhebung der soziodemographischen Merkmale der Kommunen: Größe, Anteil an Personen mit Migrationshintergrund, Altersdurchschnitt, Deprivationsindex	Teilnehmende/interessierte Kommunen und deprivierte Vergleichskommunen *n* = 171 Kommunen
2	Webbasierte Inhaltsanalyse	Online-Projektdatenbanken, Internetseiten von Kommunen	Erhebung der gesundheitsförderlichen Aktivität	Ausgewählte Kommunen der drei Untersuchungsgruppen *n* = 22 Kommunen
3	Qualitative Dokumentenanalyse	Gesprächsprotokolle, Schriftverkehrsdokumente	Erhebung von Einflussfaktoren auf die Teilnahme an Gesundheitsförderungsprojekten	„Neue“ teilnehmende Kommunen und interessierte Kommunen *n* = 37 Kommunen
